# Shape, form, function and *Leishmania* pathogenicity: from textbook descriptions to biological understanding

**DOI:** 10.1098/rsob.170165

**Published:** 2017-09-13

**Authors:** Jack Sunter, Keith Gull

**Affiliations:** 1 Department of Biological and Medical Sciences, Oxford Brookes University, Headington Campus, Oxford OX3 0BP, UK; 2 Sir William Dunn School of Pathology, University of Oxford, Oxford OX1 3RE, UK

**Keywords:** morphology, *Leishmania*, pathogenicity, parasite

## Abstract

The shape and form of protozoan parasites are inextricably linked to their pathogenicity. The evolutionary pressure associated with establishing and maintaining an infection and transmission to vector or host has shaped parasite morphology. However, there is not a ‘one size fits all’ morphological solution to these different pressures, and parasites exhibit a range of different morphologies, reflecting the diversity of their complex life cycles. In this review, we will focus on the shape and form of *Leishmania* spp., a group of very successful protozoan parasites that cause a range of diseases from self-healing cutaneous leishmaniasis to visceral leishmaniasis, which is fatal if left untreated.

## 1. Shape and form of *Leishmania*

Like many protozoan parasites, *Leishmania* have a digenetic life cycle involving both a mammalian host and an insect vector. *Leishmania* parasites exhibit a variety of different cell morphologies and a number of cell types (developmental forms) that are adapted to either the host or the vector. As seen with other parasites such as *Plasmodium* and trypanosomes, some of these developmental forms are proliferative, whereas others are quiescent and pre-adapted for transmission to the next host [1–4]. Much of the interpretation of cellular form and function in *Leishmania* species is derived from the more studied basic cell biology of trypanosomes. While this is a natural transfer of knowledge, one has to remain vigilant to the fact that unrecognized differences may exist between the two pathogen systems, even in their basic biology.

**Figure 1.**
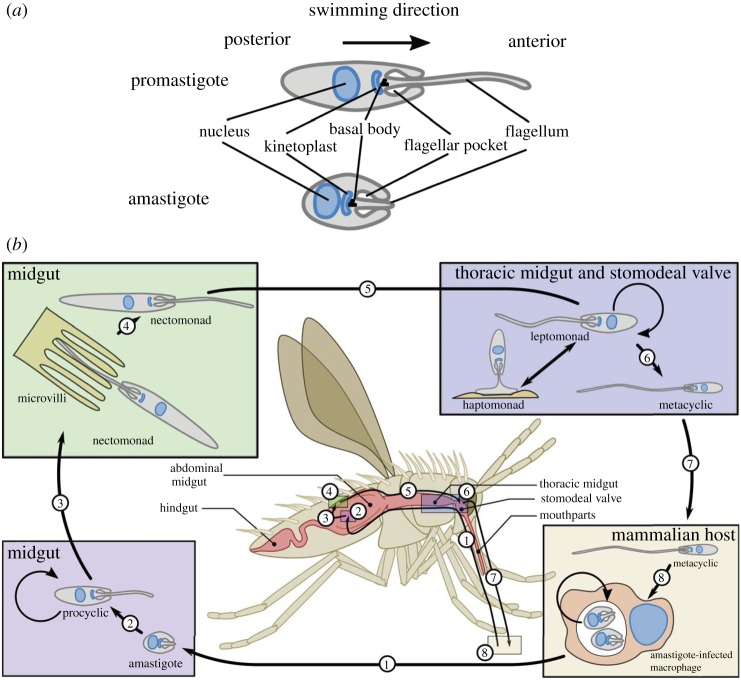
Schematic of promastigote and amastigote morphologies and the *Leishmania* life cycle with the different cell types highlighted. (*a*) Promastigote and amastigote morphologies aligned along the posterior anterior axis with key structures in the cells indicated. (*b*) Cartoon of the current understanding of the *Leishmania* life cycle with critical events and different cell types highlighted. A sand fly takes a blood meal from an infected mammalian host and ingests a macrophage containing *Leishmania* amastigotes. Once in the sand fly midgut, the amastigotes differentiate into procyclic promastigotes. Next, the procyclic promastigotes become nectomonad promastigotes, which escape the peritrophic matrix and then attach to the microvilli in the midgut before moving to the thoracic midgut and stomodeal valve where they differentiate into leptomonad promastigotes. Here, the leptomonad promastigotes differentiate into either haptomonad promastigotes which attach to the stomodeal valve or metacyclic promastigotes that are the mammalian infective form, which are transmitted when the sand fly next takes a blood meal. Proliferative stages are indicated by a circular arrow.

*Leishmania* have two major different cell morphologies, exemplified by the promastigote morphology in the sand fly and the amastigote morphology in the mammalian host (figure 1*a*). The basic cellular architecture is however conserved between the two *Leishmania* cell shapes and is defined by cross-linked sub-pellicular corset microtubules. This array is maintained throughout the cell cycle, so cell division relies on the insertion and elongation of microtubules into the existing array. Housed within the cell are the nucleus and a set of single-copy organelles such as the mitochondrion and the Golgi apparatus. Anterior of the nucleus is the kinetoplast, the mass of concatenated mitochondrial DNA which is directly connected to the basal body from which the flagellum extends [5–8]. At the base of the flagellum is an invagination of the cell membrane forming a vase-like structure called the flagellar pocket, which is important in these parasites as it is the only site of endocytosis and exocytosis and is hence a critical interface between the parasite and its host environment [9].

In essence, the *Leishmania* cell is constructed from a series of modular units such as the flagellum, basal body–mitochondrial kinetoplast unit and a Golgi–flagellar pocket neck unit [8]. These modular units are then positioned relative to each other to give rise to the different cell morphologies observed [10,11]. The key to defining the dynamic shape and form of this parasite is therefore to understand the morphogenesis of these different individual modular units and their positioning relative to each other.

Cellular morphology of the *Leishmania* parasites is very precisely defined by cell shape, flagellum length, kinetoplast/nucleus position and ultrastructural features, and therefore has been traditionally used to define the cell forms observed. In some cases, though, these morphological descriptions of cell forms have entered the literature as defining specific cell types in the life cycle. However, there are currently few molecular markers to assist in defining life cycle forms more precisely and there is a need, therefore, for care and caution when defining cell types solely on the basis of cell morphology.

## 2. Defining diversity: different species, different diseases, different cells in the vector and host

Different species of *Leishmania* parasite cause disease in humans, with the different species often grouped together depending on whether they emerged in the old world or the new world (table 1) [12] and on the nature of the pathology (cutaneous, mucocutaneous or visceral leishmaniasis) [12,13]. It is important to remember that this is not just a disease of humans and that *Leishmania* will infect other mammals, creating a zoonotic reservoir that has serious implications for disease control [14]. The sand fly vector adds a further layer of complexity: there are many species capable of carrying the *Leishmania* parasite; however, there are often specific relationships whereby some sand fly species are capable of transmitting only a single or limited number of *Leishmania* species (table 1) [15].

**Table 1.** The vector, disease and origin of a range of different *Leishmania* species. Adapted from Bates [12].

species	sand fly vector	disease	old world or new world
*L. major*	*Phlebotomus duboscqi* *Phlebotomus papatasi* *Phlebotomus salehi*	cutaneous	old world
*L. mexicana*	*Lutzomyia olmeca olmeca*	cutaneous	new world
*L. braziliensis*	*Lutzomyia wellcomei* *Lutzomyia complexus* *Lutzomyia carrerai*	mucocutaneous	new world
*L. donovani*	*Phlebotomus argentipes* *Phlebotomus orientalis* *Phlebotomus martini*	visceral	old world
*L. infantum*	*Phlebotomus ariasi* *Phlebotomus perniciosus* *Lutzomyia longipalpis*	visceral	new and old world

Despite all these levels of complexities and differences, the morphology of the different *Leishmania* species shows remarkable conservation of form as they progress through their life cycle. Cells with an amastigote or promastigote morphology look dramatically different, but they retain the same basic cell layout with the kinetoplast anterior to the nucleus and a flagellum extending from the basal body (figure 1*a*) [5,6]. An amastigote morphology is typified by a smaller and more spherical cell body with a short immotile flagellum that barely emerges from the flagellar pocket and is potentially more focused on sensory functions [16,17]. Conversely, the promastigote morphology is defined by an elongated ovoid cell body with a long motile flagellum extending out of the flagellar pocket that provides propulsive force likely responsible for facilitating the traverse through the sand fly digestive tract [18].

## 3. Promastigote to amastigote transition

When a *Leishmania*-infected sand fly takes a blood meal, metacyclic promastigotes are deposited into the site of the bite (figure 1*b*). The damage caused by the sand fly results in the recruitment of macrophages to the bite site, and these are the cells which *Leishmania* infects and resides in allowing them to persist in the host [19–21]; however, there is minimal evidence showing where the interaction between *Leishmania* and macrophages occurs in the host. Metacyclic promastigotes are highly motile cells, and *Leishmania* are able to migrate through a collagen matrix [22]. It is therefore possible that phagocytosis of *Leishmania* may occur at locations far removed from the bite site. Moreover, perhaps the spectrum of disease caused by *Leishmania* from cutaneous to visceral is reflected in the ability of the parasite to invade the host beyond the bite site either directly or via infected macrophage movement.

There are conflicting reports in the literature over the polarity of the interaction and uptake between *Leishmania* and the macrophage, with some suggesting it occurs via the flagellum first but others showing that it predominantly occurs via the cell body or from both ends [23–25]. Given the flagellum-first movement exhibited by these metacyclic promastigotes, the tip of the flagellum is likely to be the first part of the cell to interact with the macrophage. In the closely related parasite *Trypanosoma brucei*, the flagellum tip has a number of proteins including receptors that localize exclusively to this region [26,27]. There is therefore potential for a differentiated membrane domain at the flagellum tip to be primed for the collision with the macrophage, which would initiate a series of signalling events leading to the successful uptake and differentiation of the parasite. However, whichever orientation the *Leishmania* is engulfed in, the parasite ends up with its flagellum pointing towards the periphery of the macrophage [24,25]. The continued movement of the flagellum within the macrophage results in plasma membrane damage and lack of integrity, which promotes lysosomal exocytosis potentially altering the composition of the parasitophorous vacuole, thereby increasing the chances of the parasite successfully infecting the macrophage [25].

Once inside the macrophage, the promastigote differentiates from a motile promastigote form, which has a long flagellum and an elongated cell shape, to an amastigote form that has a short flagellum with only a small bulbous tip extending beyond a now more spherical cell body. This is a dramatic change in cell shape and results in a minimized cell surface to volume ratio, hence reducing the area over which the cell is exposed to the harsh environment of the parasitophorous vacuole, and also a likely reformatting of flagellum use [28–30].

The most striking difference between the amastigote and promastigote forms is the change in the flagellum from a long motile flagellum with a 9 + 2 axoneme to a short non-motile flagellum with a 9 + 0 (9v) axoneme arrangement [17]. Wheeler *et al.* [17] have studied this specific aspect of the differentiation process in detail, and have shown that this change in flagellum structure involves either (i) disassembly of the existing long flagellum and removal of its central pair, hence collapsing it down to a short 9v axoneme, or (ii) the assembly of a short new flagellum, lacking a central pair and exhibiting a 9v axoneme. The latter mechanism is interesting as the pro-basal body which assembled that 9v axoneme would have assembled a 9 + 2 axoneme if that cell had remained in promastigote culture, showing the ability of the pro-basal body to switch between constructions of either type of axoneme.

Given that there are examples of organisms that are able to completely lose and reform their flagellum during their life cycle, the continued presence of the flagellum in amastigotes suggests that it has an important function for the parasite within the macrophage [30–32]. The most commonly postulated function for this flagellum is a sensory role because the 9v axonemal architecture is structurally similar to that found in mammalian primary cilia and the tip of the flagellum in the *Leishmania* amastigote is often found in close contact with the parasitophorous vacuole membrane [16,33]. The *Leishmania* parasite, via its flagellum, could potentially sense the ‘health’ of its host macrophage by assessing key metabolites such as the adenosine nucleotides, for instance. If the macrophage is ‘healthy’, the parasite may divide, but if the macrophage is ‘unhealthy’, the parasite may decide not to divide as the macrophage may be about to die and lyse, thereby releasing the parasite into a new environment. Specific checkpoints are therefore likely to exist and be applied in the *Leishmania* cell cycle at the G1 to G0/S boundaries. Moreover, the release of the parasite is unlikely to be a ‘passive’ process but instead be driven by the parasite itself. This is an area of interest and importance where the specific cell biology of a possible parasite cell-cycle-related release has not been addressed.

In addition to the dramatic change in the flagellum structure during differentiation into the amastigote form, there is also a large restructuring of the flagellar pocket and neck region that is associated with changes in the localization of the flagellum attachment zone proteins [7]. A surprising consequence of this rearrangement is the closing of the flagellar pocket neck so that there is no observable gap between the flagellum and flagellar pocket neck membrane [7]. The dogma for the flagellar pocket in this and related parasites is that it is the only site of exocytosis and endocytosis in the cell and hence is a major interface between the parasite and its hosts. However, if the flagellar pocket is closed off at the neck, how will this affect this important interface? Is this closure dynamic and more akin to a valve-like operation? Does the limited access to the flagellar pocket reduce the ability of the parasite to take up macromolecules from its surroundings? The growth rate of axenic amastigotes has been measured by heavy water labelling and was shown to be much slower than that of promastigotes [34], and in addition amastigotes have a much smaller cell volume than promastigotes, resulting in a concomitantly smaller metabolic load. Taken together, the slow growth rate and smaller metabolic load may reflect a reduced rate of macromolecule uptake into the cell. However, the slow growth rate might be the result of an evolutionary pressure not to overwhelm the host's immune system, thereby allowing the host to survive for longer, the parasite to proliferate for longer and so increasing the chance of parasite transmission to a sand fly.

The closing of the flagellar pocket neck is also likely to be a consequence of protecting a potentially vulnerable domain of the cell from the environment within the parasitophorous vacuole, which is acidic and full of proteases [28,29]. The reduced access to the flagellar pocket due to the reduction in the space between the neck and flagellum membranes in the amastigotes is the probable explanation for lytic high-density lipoprotein containing trypanolytic factor being unable to lyse amastigotes in the acidic parasitophorous vacuole, yet being able to kill metacyclic promastigotes, which have a more accessible flagellar pocket [35]. It will be interesting to compare the morphology of the *Leishmania* amastigote flagellar pocket with that of the intracellular amastigote of the closely related organism *Trypanosoma cruzi*, as this parasite escapes from the parasitophorous vacuole and proliferates in a completely different environment, the cytoplasm of its host cell [36].

There are two distinct types of parasitophorous vacuoles that develop in the infected macrophages, which correlate with the species of *Leishmania*. Infection with some species such as *L. amazonensis* produces large multiple-occupancy parasitophorous vacuoles (type II vacuoles) that contain multiple amastigotes, whereas other species such as *Leishmania major* produce small, tight-fitting, single-occupancy parasitophorous vacuoles (type I vacuoles) that surround a single amastigote parasite [37,38]. Co-infections with *L. amazonensis* and *L. major* in a single macrophage showed that fusion of the two types of parasitophorous vacuole, either large containing *L. amazonensis* amastigotes or small containing a *L. major* amastigote, did not occur. This suggests that the parasitophorous vacuole is modified to the specific requirements of each species, precluding successful fusion of the different parasitophorous vacuole types [37].

Interestingly, despite the differences observed in parasitophorous vacuole types, the cellular organization and layout of the amastigotes of different species are well conserved [39]. However, there are some observable differences between amastigotes of different species, with *L. mexicana* amastigotes being around 50% larger in mean diameter than those of *L. braziliensis* and *L. donovani* [40]. This size difference may have implications on the pathology caused by the different *Leishmania* species, but there is no simple relationship between this or other features to pathology type. Indeed, as another example, ultrastructural studies on the amastigotes of *L. tropica* and *L. donovani* have revealed a distinct posterior invagination termed a ‘cup’ or ‘posterior invagination’ [41,42]. The authors suggested that this may be an alternative site of exo/endocytosis in these cells. To the best of our knowledge, this structure has not been found in any other species of *Leishmania* amastigotes. As *L. tropica* causes cutaneous leishmaniasis and *L. donovani* causes visceral leishmaniasis, this morphological adaptation again appears not to be linked to the disease pathology. On balance, therefore, while differences between promastigote and amastigote are likely very significant to the host/vector relationships, the overall similarity in amastigote morphology between the different species suggests that no simple link between morphology and disease pathology exists. Instead, either subtler features or the possession and expression of various potential virulence factors produced by the different species, such as the A2 protein, may have a greater import for the spectrum of disease [43,44].

A distributed skin population of *Leishmania*-infected macrophages has recently been observed by Doehl *et al.* [45]. This distributed, circulating population of infected macrophages will help to ensure efficient transmission of *Leishmania* parasites from the host to the sand fly as a splenic infection is not accessible to sand flies and sand flies are unlikely to bite exclusively at the actual lesion site in a localized cutaneous infection. Moreover, these infected macrophages may contain a potentially different form of *Leishmania* amastigote that is primed to survive in the sand fly midgut when taken up in the blood meal, mirroring the transmissibility of the metacyclic promastigote cell type in the sand fly or the stumpy form of the African trypanosomes [12,30,46].

## 4. Amastigote to promastigote transition

After ingestion by the sand fly and release from the macrophage, the amastigote begins to differentiate into a motile promastigote form. The exact cues for differentiation have yet to be established but are likely to be a combination of the change in temperature and pH akin to other parasites and as seen for *Leishmania* as it differentiates from a promastigote to an amastigote in the parasitophorous vacuole [47]. However, there may also be a requirement for the presence of a specific chemical trigger to ensure that differentiation occurs only in the vector and not in the host; for example, *Plasmodium* requires xanthurenic acid, a mosquito eye pigment precursor, to differentiate [48]. Temperature alone is unlikely to be the sole trigger for differentiation, as the macrophage will experience a range of temperatures as it circulates through the body, but it may act to sensitize the amastigote to the other differentiation cues as found with *T. brucei* [49].

The *in vitro* differentiation of *L. amazonensis* amastigotes into promastigotes has been studied in detail by microscopy [50]. The first visible step in this process is the elongation of a motile flagellum, which occurs before cell division, and after this first cell division both daughter cells had a motile flagellum [50]. On maturation, the pro-basal body in the parental cell was therefore able to assemble a 9 + 2 motile axoneme, yet if this same cell had remained in the macrophage the same pro-basal body would have assembled a 9v axoneme, again demonstrating the multipotency of the pro-basal body in *Leishmania* [17]. These results are complementary with those of Wheeler *et al.* [17] and show that pro-basal bodies are able to assemble either a 9 + 2 or a 9v axoneme independently of whichever axoneme type the mother basal body had produced [17,50].

During differentiation into a promastigote form, the cell shape also begins to change from the spherical amastigote to a more elongated ovoid shape. In concert with the changes that occur to the overall shape of the cell body, the organization of the flagellum attachment zone and flagellar pocket changes. Specifically, the neck region of the flagellar pocket becomes more open, reflecting a reversal of the process that occurred during differentiation into the amastigote form [7]. This opening of the neck may allow easier access to the flagellar pocket, enabling the uptake of large macromolecules and also potentially affecting the motility of the parasite.

## 5. Roles of the *Leishmania* flagellum in the sand fly

The flagellum has multiple potential roles in enabling the *Leishmania* parasites to successfully establish and maintain an infection in the sand fly. There are three potential key functions for the flagellum in the sand fly, which we discuss in the following sections:

(i) motility to escape the peritrophic matrix and migrate to the foregut,
(ii) attachment to the midgut microvilli and stomodeal valve, and
(iii) potential sensory functions.

### 5.1. Motility

The ingestion of a blood meal by a sand fly causes numerous changes to the sand fly, including the creation of a peritrophic matrix from chitin and glycoproteins that encases the blood meal separating it from the midgut epithelium. After approximately 4 days, the remaining undigested blood meal and surrounding peritrophic matrix are defecated out by the sand fly [51,52]. The *Leishmania* promastigotes therefore need to escape from the peritrophic matrix before defecation occurs. Moreover, for successful transmission to a mammalian host, the *Leishmania* parasites need to colonize the stomodeal valve region of the sand fly and so migrate from the midgut towards the mouthparts; active flagellar motility presumably assists in both these processes.

### 5.2. Attachment

The loss of peritrophic matrix integrity allows the *Leishmania* parasites to escape the endotrophic space [53]; these cells then attach to the epithelium of the midgut by inserting their flagella between the microvilli, which helps prevent the parasites being expelled from the sand fly during defecation. This attachment is not accompanied by any observable morphological changes to the *Leishmania* cell [54]. Evidence suggests that attachment is mediated through specific glycoprotein–lectin interactions, providing a potential mechanism by which the vector–parasite specificity is determined. The surface coat component lipophosphoglycan (LPG) was believed to be crucial for this interaction as *L. major* cells deficient in LPG synthesis were unable to attach to the sand flies *Phlebotomus papatasi* and *P. duboscqi* [55,56]. However, recent work has shown that this *L. major* mutant is able to successfully infect other sand fly species such as *P. arabicus*, *P. argentipes*, *P. perniciosus* and *Lutzomyia longipalpis*, and moreover a *L. mexicana* mutant that is unable to synthesize LPG is also able to fully develop within *Lutzomyia longipalpis* [56–58]. Clearly, LPG is important for interactions between some *Leishmania* species and sand fly species, but it is not the universal determinant of these interactions.

The second site of *Leishmania* attachment in the sand fly is at the stomodeal valve, where a specialized cell type called the haptomonad is observed attached to the cuticle lining of the valve by hemidesmosomal structures that are found in the enlarged tip of a relatively short flagellum [54]. These hemidesmosomal structures are reminiscent of those observed for the attached epimastigote forms of trypanosomes, and it is likely that attachment to the insect vector via such structures will be a universal feature of kinetoplastids [59,60]. Currently, the biochemical identity of these structures is cryptic, but given their importance in many kinetoplastid species the discovery of the molecular components will be of great interest [60]. The strong attachment presumably stops the *Leishmania* haptomonad cell type being passed to the mammalian host when the sand fly feeds and may therefore have a role in maintaining a long-term infection in the sand fly and/or asymmetric divisions [61].

### 5.3. Sensory

After escaping the peritrophic matrix, the parasites then migrate forward through the sand fly to colonize the thoracic midgut. Successful colonization and transmission of *Leishmania* are dependent on the sand fly taking a sugar meal after the blood meal, and it is an enticing hypothesis that the *Leishmania* are able to navigate the sugar gradient along the gut to enable colonization of the sand fly foregut. The molecular components required for chemotaxis are present in *Leishmania* with carbohydrate receptors found on the cell surface and a flagellum capable of performing different beat structures, which enable the cell to move forward and re-orientate itself. Furthermore, *in vitro* experiments have shown that *Leishmania* are able to respond to a change in sugar concentration [62–64].

Table 2 outlines publications in which morphology and/or motility have/has been altered by mutational analysis in *Leishmania* species [18,65–78]. Clearly, some morphology mutants will have catastrophic effects on cell division and as such are not that useful for assessing links to pathogenicity and development. Comparison with work in *T. brucei* shows that subtle RNAi knockdowns (available now as a technology in *L. braziliensis*) can provide dramatic changes in cell architecture [11,79,80]. One lesson from this is that subtle differences in expression rather than absence or presence in genomes might influence virulence or pathology. The current improvements in both reverse and forward genetics in *Leishmania* will be very helpful in this area [81–84]. We have outlined above three functions of the *Leishmania* flagellum in the sand fly, but what is the actual evidence that the flagellum is required in sand flies? From table 2, we can see that there are many mutants where the function/length of the *Leishmania* flagellum has been compromised, such as through the overexpression of KIN13-2 or the loss of PFR2, but very few studies have infected sand flies with these mutants [18,65,72].

**Table 2.** Summary of morphological and/or motility mutants in *Leishmania*.

protein	gene ID	phenotype	conserved across *Leishmania* species on TriTrypDBv33	development in sand flies	pathogenicity in macrophages	pathogenicity in animals	reference
PFR2	LmjF.16.1425/1427/1430	PFR-2 knockout cells had an altered flagellar beat with a reduced swimming velocity	yes	not done	not done	not done	[65]
PFR1	LmjF.29.1750/1760/1770	both PFR1 knockout cells and PFR1/2 double knockout cells had an altered flagellar beat with a reduced swimming velocity	yes	not done	not done	not done	[66]
ARL-3A	LdBPK_290950.1	cells overexpressing a constitutively ‘active’ form of ARL-3A were immotile with short flagella, and flagellum length was inversely proportional to mutant protein expression	yes	unable to develop in sand flies	no change in macrophage infectivity	not done	[18,67]
MKK	LmxM.08_29.2320	MKK knockout cells had motile flagella, which was dramatically shorter and lacked a paraflagellar rod and also had shorter cell bodies	yes	not done	not done	not done	[68]
MPK9	LmxM.19.0180	MPK9 knockout cells had longer flagella, whereas overexpression led to a subpopulation with short/no flagella	yes	not done	not done	not done	[69]
DHC2.2	LmxM.27.1750	DHC2.2 knockout cells were immotile and had a rounded cell body. The flagellum did not extend beyond the cell body and lacked a paraflagellar rod and other axonemal structures	yes	not done	not done	not done	[70]
MPK3	LmxM.10.0490	MPK3 knockout cells had shorter flagella with stumpy cell bodies	yes	not done	not done	not done	[71]
KIN13-2	LmjF.13.0130	KIN13-2 knockout cells had longer flagella, whereas overexpression led to shorter flagella	yes	not done	not done	not done	[72]
ADF/cofilin	LdBPK_290520.1	ADF/cofilin (actin-depolymerizing factor) knockout cells were immotile with shorter flagella and a disrupted beat pattern. The cells were also shorter and wider	yes	not done	not done	not done	[73]
katanin	LmjF13.0960	cells overexpressing katanin-like homologue had shorter flagella	yes	not done	not done	not done	[74]
SMP1	LmjF.20.1310	loss of SMP1 caused a reduction in flagellum length and defects in motility	yes	not done	not done	not done	[75]
DC2	LdBPK_323050.1	DC2 knockout cells had shorter flagella with a disrupted ultrastructure and reduced motility. Moreover, the cell bodies were shorter and rounder.	yes	not done	slight increase in macrophage infectivity	not done	[76]
inhibitor of serine peptidase 1 (ISP1)	LmjF.15.0300	ISP1/2/3 triple knockout cells had longer flagella and were less motile than ISP2/3 double knockout cells. Moreover, the triple knockout had a greater number of cells with haptomonad, nectomonad and leptomonad morphologies. There was also a change in the shape of the anterior end of these cells	yes	not done	reduced survival in macrophages	not done	[77]

Overexpression of an LdARL-3A mutant in *L. amazonesis* resulted in cells that were unable to assemble a full-length flagellum and that had impaired motility [67]. When these cells were used to infect sand flies, these mutants were unable to establish an infection, likely due to their defective motility [18]. Moreover, the length of the flagellum may also be playing a role: a shorter flagellum will be less effective at intercalating between the microvilli in the midgut, giving a smaller surface area with which to interact, reducing the strength of binding and increasing the likelihood of expulsion during defecation. However, recently, a *L. braziliensis* mutant was isolated from a patient lesion, which was unable to assemble a full-length flagellum when grown under promastigote culture conditions *in vitro* [85]. The flagellum in the mutant cells only just emerged from the flagellar pocket, and surprisingly these mutants successfully infected sand flies, though the infections were analysed only up to 4 days after feeding, so the ability of this mutant to maintain an infection over the longer term is unknown. The exact role(s) of the *Leishmania* flagellum within the sand fly has yet to be fully elucidated, and it would therefore be useful to analyse the potential of a range of flagellum mutants of *Leishmania* to establish and maintain an infection in sand flies.

## 6. Promastigote transitions

**Figure 2.**
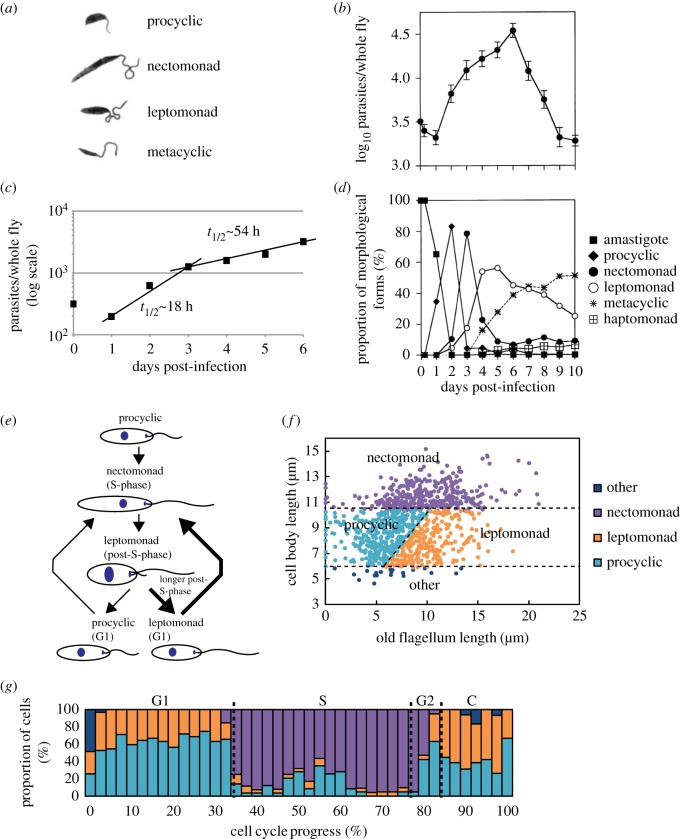
Development of *Leishmania* in the sand fly digestive tract. (*a*) Illustrations of the major promastigote morphologies observed in the sand fly during a *Leishmania* infection. (*b*) *Leishmania* cell number per sand fly during a typical sand fly infection over the course of 10 days. (*c*) Cell density from (*b*) was re-plotted and the doubling times calculated for the early and late infection stages. (*d*) Analysis of the proportions of different cell types observed during a sand fly infection. (*a*), (*b*) and (*d*) are reproduced with permission from Rogers *et al.* [86]. (*e*) Schematic of *Leishmania* cell cycle with the corresponding cell types shown above. (*f*) Correlation of flagellum and cell body length from three independent *L. mexicana in vitro* cultures analysed at different cell densities. The data were then subsequently classified into the different promastigote morphologies [86]. (*g*) Proportion of cells with different promastigote morphology by cell cycle progress. The cell cycle progress of the cells used in the analysis for (*f*) was calculated based on their cell length and DNA content and then combined with the promastigote morphology classification from (*f*) [87,88]. Dotted lines indicate transitions between cell cycle stages (C, cytokinesis). (*e*) and (*f*) are reproduced with permission from Wheeler [89].

In addition to the overarching promastigote and amastigote morphologies in the sand fly vector and mammalian host, respectively, there are variations of promastigote morphologies found such as the procyclic and metacyclic promastigotes in the sand fly. The reported shape of these different forms can be extreme, and this has led to them being defined as different cell types (developmental forms) rather than just different transition morphologies. Currently, there are four major cell types identified in the sand fly based on the length/width of the cell body and flagellum [86] (figure 2*a*):

(i) procyclic promastigote: cell body length between 6.5 and 11.5 µm with the flagellum shorter than cell body,
(ii) nectomonad promastigote: cell body longer than 12 µm,
(iii) leptomonad promastigote: cell body length between 6.5 and 11.5 µm with the flagellum longer than cell body, and
(iv) metacyclic promastigote: cell body less than 8 µm long and 1 µm wide with a flagellum longer than the cell body.

These four cell types are thought to represent a developmental sequence with specific precursor–product relationships between them (figure 1*b*). Briefly, the procyclic promastigote occurs within the blood meal; the nectomonad promastigote is observed as the peritrophic matrix breaks down and moves towards the foregut where it becomes the leptomonad promastigote before differentiating into either an infective metacyclic or a haptomonad promastigote [86,90]. In addition to these four major forms, the attached haptomonad promastigote and the paramastigote are also observed in the sand fly but at a much lower frequency [86]. All these forms have the same basic promastigote cell architecture with the kinetoplast (mitochondrial DNA) anterior to the nucleus and the flagellum extending from the anterior end of the cell with a shallow flagellar pocket, apart from the paramastigote, in which the kinetoplast is positioned next to the nucleus.

The numbers and timings of the various different promastigote cell types in sand flies have been studied by Rogers *et al.* [86] (figure 2*a*–*d*). In their experimental system, they calculated that each sand fly could ingest approximately 3200 amastigotes in a 1.6-µl bloodmeal. After ingestion, there was an initial drop of parasites over the first day to approximately 2500 and then the population grew over the next 5 days and peaked at approximately 35 000 parasites per fly, which means that approximately four doublings of the parasite population occurred (figure 2*b*). Initially, the growth of the cells was relatively quick with a doubling time of approximately 18 h in the period from 1 to 3 days post-infection. The growth rate then slowed and the doubling time dropped to approximately 54 h from days 3 to 6 (figure 2*c*). After day 6 there was a rapid decrease in the number of parasites present, with only a few thousand left by day 10. The reasons for the dramatic fall in parasite number are not known, but it may be due to the exhaustion of nutrient supplies or an immune response by the sand fly.

At each day post-infection, Rogers *et al.* analysed the different promastigote cell types present in the sand fly and showed that the growth and division of *Leishmania* appears initially relatively synchronous as clear successive peaks of different promastigote cell types are observed (figure 2*d*) [86]. Over the first 2 days of infection, in addition to a large increase in overall parasite numbers there was a rapid drop in the proportion of amastigotes with a concomitant increase in the proportion of procyclic promastigotes observed. Taken together, this means that the differentiation of amastigote into procyclic promastigote includes both direct cell differentiation and division-directed differentiation matching the *in vitro* differentiation [50]. After the procyclic promastigote peak, there is a peak of nectomonad promastigotes followed by leptomonad promastigotes, which then drops in proportion as the quiescent metacyclic promastigotes begin to dominate the infection.

The other consistently observed cell types of *Leishmania* in the sand fly are the attached haptomonad promastigote and the paramastigote. Where these forms have been quantified, the paramastigote was rarely seen and did not account for more than 2% of cells observed [86]. A possibility exists that it could be an aberrant division product, where the kinetoplast has ended up next to the nucleus. The position in the developmental cycle of the attached haptomonad promastigote, which is observed at low numbers and occurs in the later stages of the infection, is unclear, with it potentially deriving from a leptomonad promastigote (figure 1*b*) [86]. This cell type is rarely seen in division, which suggests that the attachment to the cuticle may be reversible and a cell may stochastically attach and detach or that it divides very slowly.

The synchrony of the appearance of the different promastigote cell types identified by Rogers *et al.* in the sand fly is intriguing as *in vitro* synchronized cell cultures rapidly lose their synchronicity, yet here it is retained for approximately four cell doublings (figure 2*d*). It is possible that the synchrony of cell division is set by the entry into proliferation as the amastigotes begin to differentiate and divide, but perhaps, there are other elements involved such as environmental conditions. The rapid proliferation of *Leishmania* parasites in the sand fly has a large effect on the nutrient levels. Might the change in nutrients create the synchrony of the different cell types observed? This would be reminiscent of diauxic growth observed in bacteria where if both glucose and galactose are present the bacteria preferentially metabolize the glucose. Once that is exhausted they switch to lactose, but this switch is accompanied by a pause in cell growth as the metabolism of the cell is reprogrammed [91,92]. Recent transcriptomic data from Inbar *et al.* [93] show that there is a drop in the level of mRNA of glucose-metabolism-related genes with an increase in the level of mRNA of amino acid transporter genes as the parasite switches from a procyclic promastigote to a nectomonad promastigote cell type.

## 7. Life cycle and cell cycle

An added complication to the analysis of the different promastigote cell types comes from the recent work on the morphological changes observed during the cell cycle of *in vitro* cultured promastigotes [87,94]. This has demonstrated that as a promastigote proceeds through the cell cycle it undergoes a doubling and then halving of its cell length. In addition, unlike for many other organisms, the two daughter cells produced by *Leishmania* promastigote cell division are different. One daughter will inherit the old and therefore longer flagellum and the other will inherit the new and therefore shorter flagellum, so promastigote cell division can generate two daughter cells with dramatically different flagellum lengths (figure 2*e*). If the morphological definition of procyclic, nectomonad and leptomonad promastigote cell types as defined by Rogers *et al.* is applied to the cells observed in culture, all three cell types are found (figure 2*f*,*g*) [86,89].

— A nectomonad promastigote looks similar to a cell in S-phase.
— A procyclic promastigote looks similar to a cell either in G1 or post-S-phase that inherited the new, short flagellum.
— A leptomonad promastigote looks similar to a cell in the same cell cycle stages as a procyclic promastigote but which inherited the old, longer flagellum.

This highlights the problems of using morphological parameters to define cell types as the two daughter cells of an *in vitro* promastigote division can be differentially classified as either a procyclic or a leptomonad cell type. It therefore also emphasizes the need for independent cell markers for the different life cycle cell types.

It is possible that the proportion of cells produced after division with a morphology that would define them as either a procyclic or a leptomonad cell type is influenced by the time the parental cell spends in G2, as this is the time during which the elongation of both the old and new flagella occurs (figure 2*e*). For example, if a cell spends sufficiently long in G2, then the subsequent division would generate two leptomonad cell types (figure 2*e*). Through manipulation of the length of G2, it is therefore conceivable that a growing population of *Leishmania* promastigotes could become dominated by leptomonad cell types as is seen in the sand fly. Moreover, the nectomonad cell type was defined as a non-dividing stage as it was never observed to have either two kinetoplasts or two nuclei [90]. Interestingly, the promastigote cells observed in culture with a morphology that would define them as a nectomonad promastigote are predominantly in S-phase and so would have only one kinetoplast and nucleus (figure 2*g*) [89].

Life cycle development is regarded as a one-way street, and so as a parasite goes through each step it commits to differentiating into the next life cycle stage without being able to revert the previous stage. This means that there will be significant differences between parasites at different stages of the life cycle including changes to metabolism, cell-surface protein expression and also cell shape. One has to be careful with the latter, however, as these characteristics are prone to change for other reasons such as cell cycle position and metabolic state. It is therefore best to define whether a parasite has become a different cell type based on molecular markers. The recent transcriptomic analysis of *Leishmania* cells in the sand fly should help to clarify the current situation [93].

## 8. Discussion

Protozoan parasites have distinctive phases of population proliferation and differentiation associated with different proliferative cell types and transmission cell types. In turn, these are associated with movement between different ecological niches in the parasite's life cycle. A general feature of transmission stages is that they are no longer proliferative, and are irreversibly differentiated and are able to proliferate again only when they have successfully moved to the next environmental site in their life cycle. For example, in *Plasmodium*, the merozoite is the proliferative cell type in the erythrocyte and the gametocyte is the cell type that establishes the infection in the mosquito. As the differentiation into a transmission form is irreversible, this process has to be tightly regulated and will therefore be influenced by the parasite's host environment [1]. Hence, parasites need to closely monitor their environment so that they can respond in the most appropriate manner, which could be either continued proliferation or a switch to a differentiation programme to prepare for transmission.

Furthermore, the decision point for differentiation must be integrated into and coordinated with the cell cycle. This implies that there is a cell-cycle-based checkpoint at which a cell must decide either to divide or to differentiate into the next developmental stage, and this decision will be based on the integration of parasite internal and external cues [1]. For *Giardia* encystation to occur, the cells need to undergo a round of DNA replication before differentiation can occur, suggesting that for this organism the decision point is during G2 [95]. Currently, identifying these development cues is an area of active research as disruption of this process has great therapeutic potential either by causing premature differentiation or by blocking the process entirely [46].

Eukaryotic parasites have evolved a dazzling array of different morphologies that are likely to be adapted to various different ecological niches. The kinetoplastid parasites are an ideal set of organisms to study and hence understand the role of cell shape and form in enabling transmission between host and vector, and also the establishment and maintenance of infection. The closely related parasites *Leishmania* spp., *T. brucei* and *T. cruzi* have each adapted to a different niche within the host and have a different vector and a different set of morphologies into which they differentiate through their life cycles. High-quality genomes are available for all three species, and comparative genomics has identified sets of genes that are unique to each of these organisms or shared between only two of them; these are likely to be involved in certain processes such as adaptations to an intracellular or extracellular lifestyle in the host [96–98].

An interesting observation highlighted by comparing *Leishmania* with *T. brucei* is that the different developmental forms of *T. brucei* observed in the tsetse fly are generated through asymmetrical divisions, which produce two different daughter cells that are dramatically different in size or have a different kinetoplast nucleus arrangement [99,100]. However, during *Leishmania* development in the sand fly, no asymmetric divisions have been reported. Have these been missed or are the different forms observed in the sand fly potentially generated through a different mechanism? During the cell cycle, a *Leishmania* cell will undergo a doubling and then halving of its cell length [87], demonstrating that an individual *Leishmania* cell has a different plasticity in cell shape and form than a trypanosome. Therefore, the different *Leishmania* forms observed in the sand fly can be generated by the modulation of the shape of an individual cell, whereas the changes that occur in trypanosome cell shape in the tsetse fly may rely on asymmetric division to generate different cell shapes.

The understanding of the biology behind the ability of *Leishmania* parasites to subvert their hosts is of great interest and a medical imperative. The parasite has specific cell types and morphologies that are clearly linked to pathogen niches, but we need to interpret these in a modern cell biological context. There is now an opportunity to revisit the textbook descriptions of shape, form and cell types using new tools and techniques. At times, we lack stringent evidence to underpin the conclusions that are generally accepted. An absence of evidence is not evidence of absence. Molecular parasitology is moving from a twentieth-century parasitology textbook description of these parasites to a modern twenty-first-century cell biology understanding of the cellular mechanisms that enable them to survive and thrive.

## Data accessibility

This article has no additional data.
