# Correction to ‘Shape, form, function and *Leishmania* pathogenicity: from textbook descriptions to biological understanding’

**DOI:** 10.1098/rsob.180134

**Published:** 2018-08-29

**Authors:** Jack Sunter, Keith Gull

*Open Biol.* **7**, 170165. (Published online 13 September 2017). (doi:10.1098/rsob.170165)

A correction is required to table 2 of our above review. All the authors gave their consent to this correction.

The correct version of table 2 is as follows:

**Table 2.** Summary of morphological and/or motility mutants in *Leishmania*.

protein	geneID	phenotype	conserved across *Leishmania* species on TriTrypDBv33	development in sand flies	pathogenicity in macrophages	pathogenicity in animals	reference
PFR2	LmjF.16.1425/1427/1430	PFR-2 knockout cells had an altered flagellar beat with a reduced swimming velocity	yes	not done	not done	not done	[66]
PFR1	LmjF.29.1750/1760/1770	Both PFR-1 knockout cells and PFR1/2 double knockout cells had an altered flagellar beat with a reduced swimming velocity	yes	not done	not done	not done	[67]
ARL-3A	LdBPK_290950.1	Cells overexpressing a constitutively ‘active’ form of ARL-3A were immotile with short flagella and flagellum length was inversely proportional to mutant protein expression	yes	unable to develop in sand flies	no change in macrophage infectivity	not done	[68,18]
MKK	LmxM.08_29.2320	MKK knockout cells had motile flagella, which was dramatically shorter and lacked a paraflagellar rod and also had shorter cell bodies	yes	not done	not done	long delay in lesion development	[69]
MPK9	LmxM.19.0180	MPK9 knockout cells had longer flagella, whereas overexpression led to a subpopulation with short/no flagella	yes	not done	not done	lesions developed slightly slower with knockout cells	[70]
DHC2.2	LmxM.27.1750	DHC2.2 knockout cells were immotile and had a rounded cell body. The flagellum that did not extend beyond the cell body and lacked a paraflagellar rod and other axonemal structures	yes	not done	not done	not done	[71]
MPK3	LmxM.10.0490	MPK3 knockout cells had shorter flagella with stumpy cell bodies	yes	not done	not done	no change in lesion development	[72]
Kin13-2	LmjF.13.0130	Kin13-2 knockout cells had longer flagella, whereas overexpression led to shorter flagella.	yes	not done	not done	not done	[73]
ADF/cofilin	LdBPK_290520.1	ADF/cofilin (actin-depolymerizing factor) knockout cells were immotile with shorter flagella and a disrupted beat pattern. The cells were also shorter and wider	yes	not done	not done	not done	[74]
Katanin	LmjF13.0960	Cells overexpressing katanin-like homologue had shorter flagella	yes	not done	not done	not done	[75]
SMP1	LmjF.20.1310	Loss of SMP1 caused a reduction in flagellum length and defects in motility	yes	not done	not done	not done	[76]
DC2	LdBPK_323050.1	DC2 knockout cells had shorter flagella with a disrupted ultrastructure and reduced motility. Moreover, the cell bodies were shorter with a more amastigote appearance	yes	not done	slight increase in macrophage infectivity	not done	[77]
Inhibitor of Serine Peptidase 1 (ISP1)	LmjF.15.0300	ISP1/2/3 triple knockout cells had longer flagella and were less motile than ISP2/3 double knockout cells. Moreover, the triple knockout had a greater number of cells with haptomonad, nectomonad and leptomonad morphologies. There was also a change in the shape of the anterior end of these cells	yes	not done	reduced survival in macrophages	not done	[78]

